# Ligation of Dectin-2 with a novel microbial ligand promotes adjuvant activity for vaccination

**DOI:** 10.1371/journal.ppat.1006568

**Published:** 2017-08-09

**Authors:** Huafeng Wang, Taek-Jin Lee, Scott J. Fites, Richard Merkhofer, Robert Zarnowski, Tristan Brandhorst, Kevin Galles, Bruce Klein, Marcel Wüthrich

**Affiliations:** 1 Department of Pediatrics, University of Wisconsin School of Medicine and Public Health, University of Wisconsin-Madison, Madison, United States of America; 2 Department of Medicine (Infectious Disease Division), University of Wisconsin School of Medicine and Public Health, University of Wisconsin-Madison, Madison, United States of America; 3 Department of Internal Medicine, University of Wisconsin School of Medicine and Public Health, University of Wisconsin-Madison, Madison, United States of America; 4 Department of Medical Microbiology and Immunology, University of Wisconsin School of Medicine and Public Health, University of Wisconsin-Madison, Madison, United States of America; University of Birmingham, UNITED KINGDOM

## Abstract

The development of vaccines against fungi and other intracellular microbes is impeded in part by a lack of suitable adjuvants. While most current vaccines against infectious diseases preferentially induce production of antibodies, cellular immunity is essential for the resolution of fungal infections. Microbes such as fungi and *Mycobacterium tuberculosis* require Th17 and Th1 cells for resistance, and engage the C-type lectin receptors including Dectin-2. Herein, we discovered a novel Dectin-2 ligand, the glycoprotein *Blastomyces* Eng2 (Bl-Eng2). Bl-Eng2 triggers robust signaling in Dectin-2 reporter cells and induces IL-6 in human PBMC and BMDC from wild type but not Dectin-2^-/-^ and Card9^-/-^ mice. The addition of Bl-Eng2 to a pan-fungal subunit vaccine primed large numbers of Ag-specific Th17 and Th1 cells, augmented activation and killing of fungi by myeloid effector cells, and protected mice from lethal fungal challenge, revealing Bl-Eng2’s potency as a vaccine adjuvant. Thus, ligation of Dectin-2 by Bl-Eng-2 could be harnessed as a novel adjuvant strategy to protect against infectious diseases requiring cellular immunity.

## Author summary

Despite several million new systemic fungal infections annually worldwide, there are no commercial vaccines available. The lack of appropriate adjuvants is one major impediment to developing safe and effective vaccines against infections with fungal pathogens. Current vaccines against infectious diseases preferentially induce protective antibodies, driven by adjuvants such as alum. While clonally-derived and adoptively transferred monoclonal antibodies may confer protection against fungi, the contribution of antibody to host defense is likely to be limited. Animal studies and clinical observations implicate cellular immunity as an essential component of the resolution of fungal infections. We found a promising adjuvant that augments cell mediated immune responses and vaccine-induced protection against fungal infection. We anticipate that our discovery will be a useful adjuvant for vaccination with non-replicating and safe subunit vaccines against many microbial pathogens that require protective cell mediated immune responses.

## Introduction

Fungal disease remains a challenging clinical and public health problem. Despite medical advances, invasive fungal infections have skyrocketed over the last decade and pose a mounting health threat in immune-competent and -deficient hosts with worldwide mortality rates ranking 7^th^, even ahead of tuberculosis [1,2]. The development of safe, effective vaccines remains a major hurdle for fungi. Critical barriers to progress include the lack of defined fungal antigens (Ags) and suitable adjuvants that together exert protective immunity. Recent strides in our understanding of fungal immunity and discovery of fungal Ags have raised the prospect that vaccines against fungi can be developed to elicit lasting protective immunity if suitable adjuvants are available.

Adaptive immunity is critical for the prevention and resolution of fungal infections. The contribution of antibodies to host defense is debated [3,4]. In contrast, Ag-specific CD4^+^ T cells play the major role in fungal resistance [4,5], as evidenced by the high incidence of life-threatening fungal infections in patients with impaired CD4^+^ T cells. CD4^+^ T cells confer resistance by secretion of T-helper 1 (Th1) and Th17 cytokines such as IFN-γ, TNF-α, GM-CSF, and IL-17A, which activate neutrophils, monocytes, macrophages and DCs for fungal clearance [3,4,6,7]. Since CD4^+^ T cells are germane to host defense against fungi, the challenge is how best to elicit these T cells.

The transition from innate to adaptive immunity is fostered by dendritic cells (DCs). These cells process and present Ag to naïve CD4^+^ T cells in the context of co-stimulatory factors (e.g. cell surface ligands and cytokines) that provide the combination of signals necessary to induce naive T cell activation and proliferation. During their interactions with DCs, naive T cells also become functionally specialized. Helper T cell polarization occurs as a result of the cytokines produced by DCs: Th1 polarization is associated with DC production of high levels of IL-12p70, and Th17 polarization is associated with DC production of IL-1β and IL-6. While vaccine Ags typically have little impact on the nature of the cytokines produced by DCs, the adjuvant can have a dramatic effect. Alum (aluminum hydroxide), which is the most commonly used adjuvant in current vaccine formulations, suppresses DC production of pro-inflammatory cytokines such as IL-12p70 [8], creating an environment that polarizes T cells towards a Th2 phenotype. Thus, a major weakness and central challenge in the field of vaccinology is the lack of adjuvants that drive Th1 and/or Th17 polarization and stimulate DCs to produce the appropriate cytokines. Pathways that can differentially activate DC cytokine profiles include toll-like receptors (TLRs), C-type lectin receptors (CLRs), co-stimulatory ligands such as CD40, and cytokine receptors.

C-type lectins are important in fungal recognition by DCs and in inducing anti-fungal Th1 and Th17 responses [9,10,11]. Dectin-1 and Dectin-2 induce Th1/Th17 cells in response to *Candida albicans* [12,13,14,15] and *Aspergillus fumigatus* [16,17,18] infection. While Dectin-1 is dispensable, Dectin-2 is requisite for the development of protective Th1 and Th17 cells and vaccine resistance against dimorphic fungi [19]. Crude fractions of mannoproteins isolated from *Malassezia pachydermatis* as well as a lipoglycan (Man-LAM) of *Mycobacterium tuberculosis* [20] have been shown to trigger Dectin-2 signaling, however they have not been evaluated as vaccine adjuvants, and glycans and lipids may be difficult to express and scale.

Here, we report the identification of a novel fungal Dectin-2 ligand from an attenuated vaccine strain of *Blastomyces dermatitidis*, Bl-Eng2. We tested whether the ligation of Dectin-2 effectively vaccinates mice against fungi. Our vaccination strategy was to ligate Dectin-2 with Bl-Eng2 and assess the adjuvant activity by combining it with the recently reported pan-fungal vaccine calnexin [21]. Fungal recombinant Bl-Eng2 was expressed and scaled efficiently, it stimulated IL-6 and IL-1β production *in vitro* and Th1 and Th17 cells *in vivo* and, when used as an adjuvant in combination with calnexin, it protected mice against pneumonia in a model of lethal pulmonary fungal infection.

## Results

**Fig 1:**
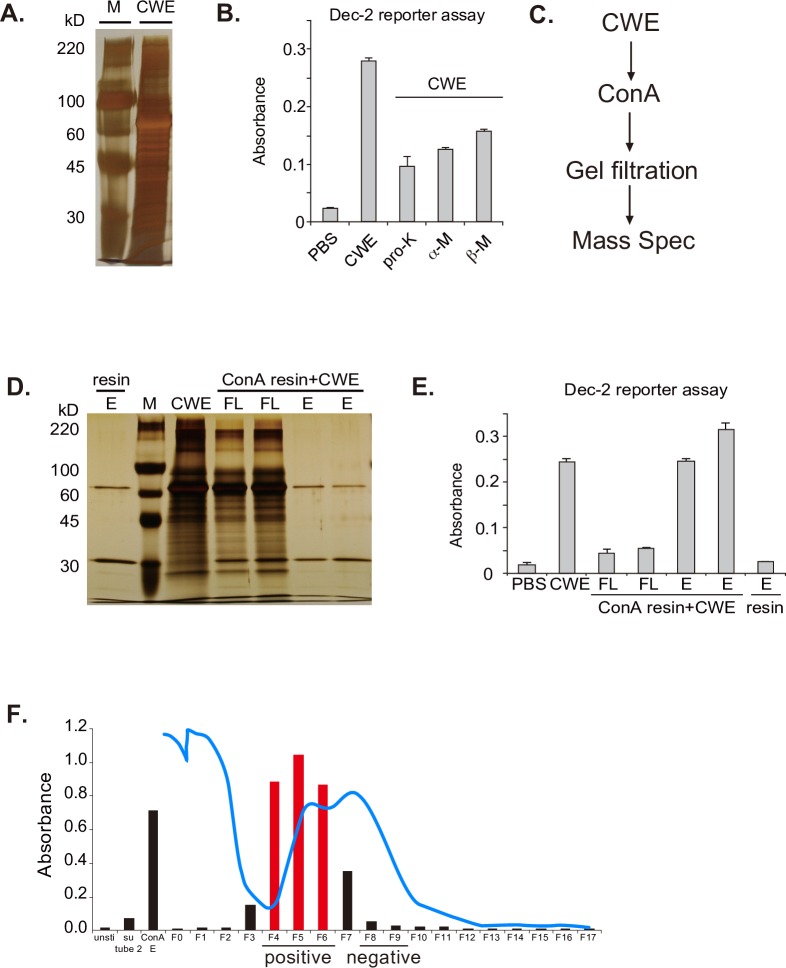
Identification of ligand activity and enrichment by ConA. **(A)** Silver-stained SDS-PAGE gel of CWE after water wash and sonication. **(B)** Dectin-2 reporter cells were stimulated with plate-coated CWE treated with or without proteinase K (pro-K), α-Mannosidase (α-M), or β-Mannosidase (β-M). After 18 h, lacZ activity was measured. Data are the mean ± SD of duplicate wells. **(C)** Flow chart of ligand enrichment and purification. **(D)** CWE was incubated with ConA resin. Flow-through (FL) and eluate were run on SDS-PAGE gel, silver stained **(E)** and analyzed for ligand activity. **(F)** ConA eluate was further separated by size exclusion using a BioLogic LP system (Biorad) and Ultro Gel ACA44 resin (Pall Corporation) at a flow rate of 1 ml/min (blue line represents the trace line of A_280_ absorption). Fractions were tested by Dectin-2 reporter cells for ligand activity. Fractions 4–6 contained most of the ligand activity and were separated by a second run over the size exclusion column (see S1C Fig).

*B*. *dermatitidis* vaccine yeast are bound by soluble Dectin-2 fusion protein and trigger NFAT signaling of Dectin-2 reporter cells [19]. Dectin-2^-/-^ mice fail to develop Ag-specific Th1 and Th17 cells or acquire vaccine resistance. We therefore sought to identify the fungal pathogen-associated molecular pattern (PAMP) that is recognized by Dectin-2. We used the NFAT-LacZ reporter cells to enrich Dectin-2 ligand activity from the vaccine yeast cell wall. We sonicated vaccine yeast, collected the water-soluble, cell-wall extract (CWE) and analyzed it by SDS-PAGE. CWE displayed a broad range of protein bands (Fig 1A) and harbored Dectin-2 ligand activity (Fig 1B). Digestion of CWE with proteinase K or endo-mannosidases reduced this activity (Fig 1B), suggesting that both protein and glycan moieties may contribute to ligand activity. To define the *Mr* of candidate proteins, we separated the CWE using a GELFREE 8100 system (S1A Fig). Fractions #5–6 ranging between 75 to 150 kDa in size contained ligand activity (S1A and S1B Fig).

**Fig 2:**
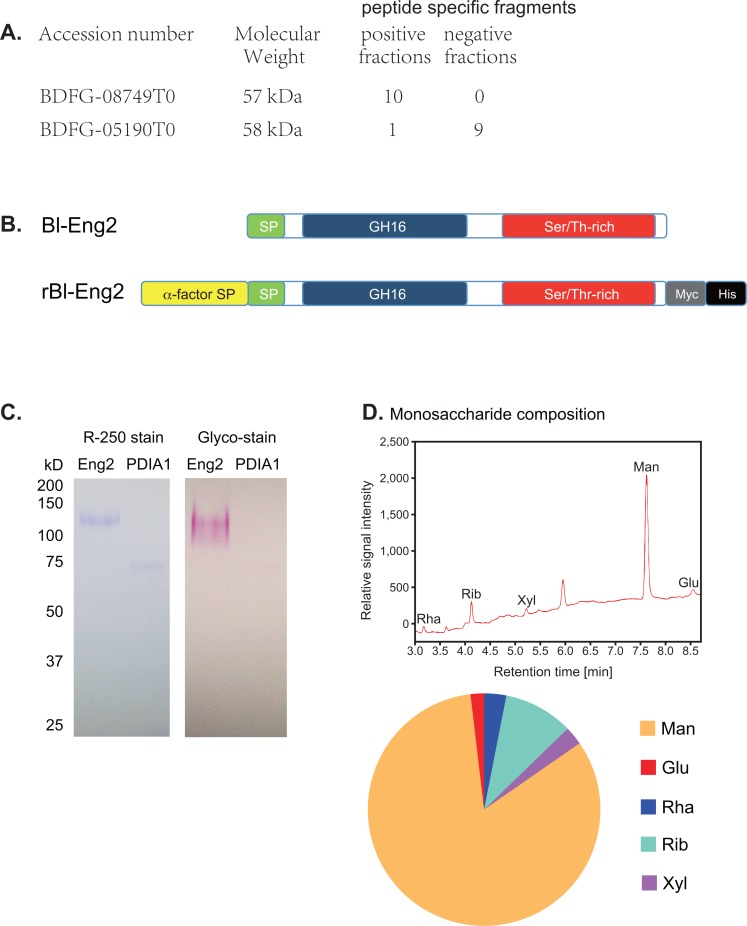
Mass spec analysis identified Bl-Eng2 as a Dectin-2 ligand candidate. **(A)** The ligand- negative and -positive fractions (#9–13 and #1–7 from S1C Fig, respectively) from the second gel filtration were analyzed by Mass spectrometry. Numbers on the right represent number of peptide specific fragments detected. **(B)** Domains of native *B*. *dermatitidis* Eng2 (Bl-Eng2) and recombinant Bl-Eng2 expressed in *Pichia pastoris*: SP denotes Signal peptide; GH16 denotes glycosyl hydrolase catalytic domain; Ser/Thr-rich domain harbors 68 potential *O*-linked glycosylation sites; and Myc and His tags are placed at the C terminus for purification. **(C)** 0.6 μg Bl-Eng2 and 0.3 μg PDIA1 were run on SDS-PAGE gel under reducing conditions and stained for protein (left) or carbohydrate (right). **(D)** Monosaccharide composition of Bl-Eng2 measured by gas chromatography (GC). GC chromatogram of the alditol acetate-derivatized monosugars of hydrolyzed Bl-Eng2 (top). Monosaccharides are labeled as follows: Rha—rhamnose, Rib—ribose, Xyl—xylose, Man—mannose, and Glu–glucose. Unlabeled peak at 5.953 min resulted from component degradation during alditol acetate derivatization. Pie diagram shows the relative contribution of monosaccharides (bottom).

To enrich and identify glycoprotein with ligand activity, we employed the lectin Concanavalin A (ConA), which binds α-D-mannose and α-D-glucose moieties, gel filtration and Mass spectrometry analysis (Fig 1C). The majority of the ligand activity was removed from CWE by a ConA resin, and the eluate was highly enriched for ligand activity (Fig 1D and 1E). The enrichment by ConA suggested that the Dectin-2 ligand(s) in CWE are mannoproteins. To further enrich ligand activity, the ConA eluate was separated by size exclusion chromatography twice, sequentially. Fractions F4-F6 after the first run contained ligand activity as determined by the Dectin-2 reporter assay (Fig 1F); F4-6 were pooled and subjected to a second separation by gel filtration. The positive fractions (F9-F13) and negative ones (F1-F7) after the second gel filtration (S1C Fig) were analyzed by mass spectrometry (Fig 2A and S2A Fig). Proteins that were more abundant in the positive vs. negative fraction were considered candidates. Among the candidates, an uncharacterized member (BDFG_08749) of the fungal endo-1,3(4)-β-D-glucanase family stood out in positive fractions (S2A Fig). The native 526-aa protein contains an 18-aa signal peptide, an N-terminal GH16 glycosyl hydrolase (GH) catalytic domain, and a C-terminal S/T-rich domain (Fig 2B and S2B Fig) that could be responsible for the strong glycosylation (Fig 2C and 2D). The GH16 catalytic domain of the endo glucanase has 60.1% similarity (identical aa and conservative substitution) (45.8% identity) and the entire glycoprotein has 45.2% similarity (28.8% identity) to the GPI-anchored endo β-1,3-glucanase Eng2 of *A*. *fumigatus* [22]. Thus, we named the protein ligand *Blastomyces*-Eng2 (Bl-Eng2). PDIA1 is a protein that was more abundant in the negative gel filtration fraction (e.g. a negative control) (Fig 2A) and showed no reporter activity and little glycosylation (Fig 2C).

### Bl-Eng2 protein is a bona-fide ligand for Dectin-2

To evaluate whether Bl-Eng2 is recognized by Dectin-2, we cloned and expressed the recombinant protein in *Pichia pastoris*. This eukaryotic expression system modifies recombinant proteins with both *O-* and *N-*linked glycosylation. Full-length Bl-Eng2 was fused to a N-terminal α-factor secretion signal and a C-terminal Myc-6×His tag (Fig 2B). Ni-NTA purified Bl-Eng2 showed a band of ~120 kDa on SDS-PAGE gel (Fig 2C), which falls within the size range determined in S1A and S1B Fig. Periodic acid-Schiff (PAS) based glyco-stain of Bl-Eng2 showed strong glycosylation (Fig 2C), which likely accounts for the discrepancy between predicted *Mr* of 57 kDa and apparent *Mr* of ~120 kDa. Gas chromatography (GC) analysis indicated that mannose is the major monosaccharide, and constitutes 82.8% in glycan mass of *Pichia*-expressed Bl-Eng2 (Fig 2D).

**Fig 3:**
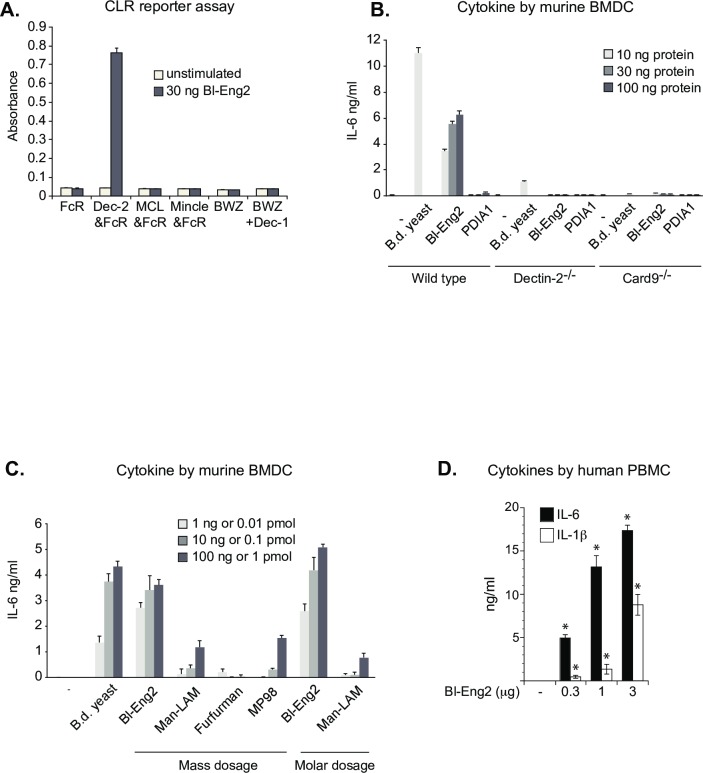
Bl-Eng2 is a bona-fide, superior Dectin-2 ligand. **(A)** *Pichia*-expressed proteins were plate-bound and tested for ligand activity using CLR expressing B3Z reporter cells expressing FcRγ chain, Dectin-2 + FcRγ, MCL + FcRγ, and Mincle + FcRγ, and BWZ cells and a subline expressing Dectin-1-CD3ζ (Dectin-1). **(B)** Supernatants from murine BMDCs (2 × 10^5^ per well) co-cultured with plate-bound Bl-Eng2 or PDIA1 were analyzed for IL-6 by ELISA. *Blastomyces* vaccine yeast (4 × 10^5^ per well) was used as positive control. **(C)** Supernatants from BMDCs (10^5^ per well) co-cultured with 1, 10, or 100 ng and 0.01, 0.1 or 1 pmol plate-bound Bl-Eng2, Man-LAM, Furfurman or MP98 were analyzed for IL-6 by ELISA. *Blastomyces* vaccine yeast (10^4^, 10^5^ or 10^6^ per well) was used as positive control. Data in A-C represent the mean ± SEM of one representative experiment of 3 independent experiments. **(D)** Bl-Eng2 induces IL-6 and IL-1β by human PBMCs. Human PBMCs were stimulated with plate-bound Bl-Eng2 for 24h and cytokines in cell culture supernatants were measured by ELISA. Data represent the mean ± SEM of 5 healthy individuals. *, p < 0.05 vs. no Bl-Eng2.

To verify Bl-Eng2 ligand activity, B3Z reporter cells expressing Dectin-2 or other distinct CLRs were incubated with recombinant Bl-Eng2. Bl-Eng2 elicited strong NFAT-lacZ signalling from Dectin-2 reporter cells, but not from the other CLR-expressing cells (Fig 3A), indicating a specific interaction between Dectin-2 and Bl-Eng2. Since *Aspergillus* Eng2 (Asp-Eng2) exhibits a high degree of similarity to Bl-Eng2 and contains a Ser/Thr-rich C terminus, we also tested whether Asp*-*Eng2 is recognized by Dectin-2. Asp-Eng2 and Bl-Eng2 were similarly recognized by Dectin-2 expressing reporter cells (S3A and S3B Fig), hence Eng2 from both fungal species are Dectin-2 ligands.

### Dectin-2 is required for Bl-Eng2 ligand activity in primary cells

To investigate whether Bl-Eng2 stimulates primary cells, we examined pro-inflammatory cytokine production from bone marrow–derived dendritic cells (BMDCs). BMDCs from wild type mice, but not Dectin-2^-/-^ or Card9^-/-^ mice, produced a strong IL-6 response when stimulated with recombinant Bl-Eng2, but not PDIA1 (Fig 3B), indicating ligand specificity for Dectin-2. Lack of stimulation of BMDCs from knockout mice also excludes the possibility of endotoxin contamination as the stimulus of IL-6 in wild type cells. Thus, *Pichia*-expressed Bl-Eng2 triggers a cytokine response *in vitro* that requires Dectin-2 and downstream Card9. These results together indicate that Bl-Eng2 appears to be a selective Dectin-2 ligand.

### Bl-Eng2 is a Dectin-2 ligand with superior capacity to elicit cytokine responses

Dectin-2 recognizes several fungi including *C*. *albicans*, *A*. *fumigatus* and *Malassezia*, which possess *N*- and *O*-linked mannan on their surface [17,18,23,24,25,26]. Thus, not surprisingly, there are two other Dectin-2 ligands described in the literature. They are Furfurman from *Malassezia spp*. [25] and Man-LAM from *M*. *tuberculosis* [20]. In addition to these ligands, by using B3Z reporter cells in the work here, we observed that MP98 from *Cryptococcus neoformans* [27] is also recognized by Dectin-2 (S3C Fig). MP98 also triggers IL-6 by BMDC in a Dectin-2- and concentration-dependent manner (S3D Fig). MP98 is a mannoprotein of *Mr* of 98 kDa with 103 Ser/Thr residues at the C-terminus that serve as potential *O*-linked glycosylation sites, and 12 putative *N*-linked glycosylation sites [27].

To begin to evaluate the relative potency of Dectin-2 ligands, we compared the ability of Bl-Eng2 and the other three Dectin-2 ligands to induce cytokine production by BMDCs. Bl-Eng2 induced the strongest IL-6 production by BMDCs when compared at equal molar and mass ratios to the other ligands (Fig 3C). These results suggest that Bl-Eng2 is relatively potent for triggering IL-6 and might be used as an adjuvant for vaccination to boost the development of Ag-specific T cell responses.

### Bl-Eng2 induces the production of IL-6 and IL-1β by human PBMCs

A suitable adjuvant for vaccine formulation should ideally stimulate human accessory cells. To test this capacity, we assessed the effect of Bl-Eng2 on the function of human PBMCs. After stimulation with plate-coated Bl-Eng2, human PBMCs from five healthy subjects produced up to 17 ng/ml IL-6 and 9 ng/ml IL-1β as measured in the cell culture supernatants by ELISA (Fig 3D). These data suggest that recombinant Bl-Eng2 has the capacity to induce the production of Th17 cell priming cytokines by human antigen-presenting cells (APC) *in vitro*.

### Bl-Eng2 promotes T cell development *in vivo* and imparts vaccine efficacy

**Fig 4:**
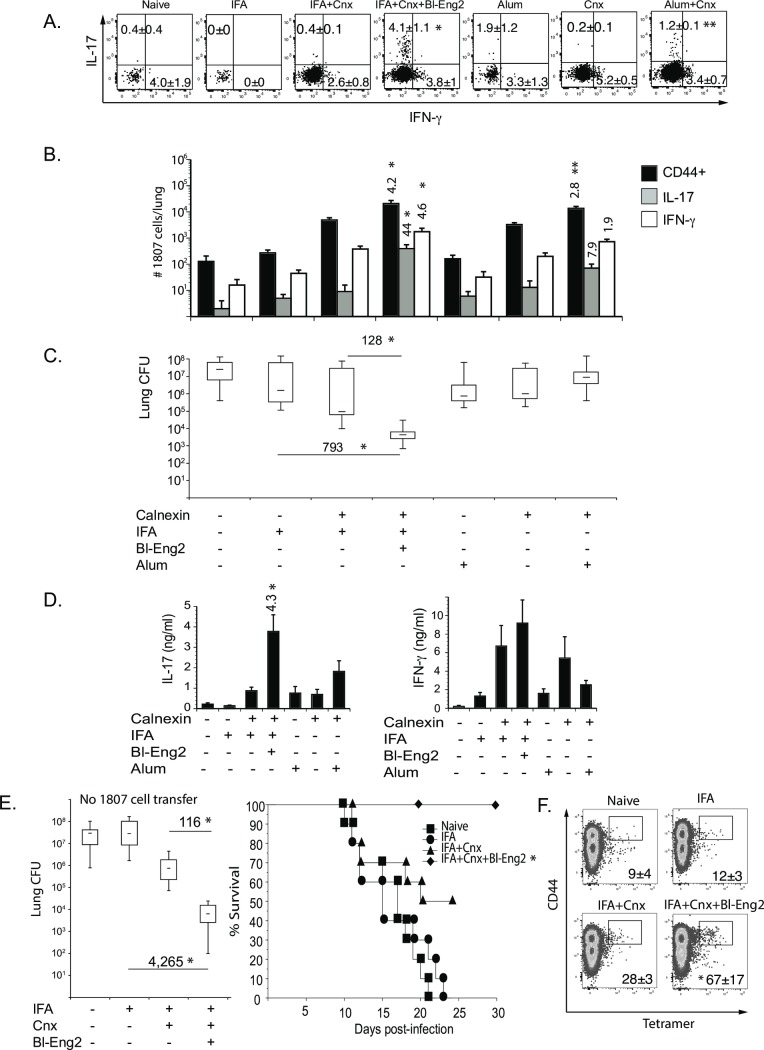
Bl-Eng2 augments CD4^+^ T cell development *in vivo*. Mice received 10^6^ adoptively transferred naïve 1807 T cells prior to vaccination **(A-D)** or no transfer **(E+F)**. Mice were subcutaneously vaccinated with 5μg calnexin and 10μg Bl-Eng2 or alum twice, two weeks apart, and then challenged intratracheally with *B*. *dermatitidis* 26199 yeast two weeks post-vaccination. At day 4 post-infection, the frequencies of IL-17 and IFN-γ producing 1807 T cells **(A)** and numbers of activated (CD44^+^) and cytokine-producing 1807 cells in the lung were enumerated by FACS **(B)**. Almost all of the 1807 T cells recruited to the lung were CD44^+^. Data represent the average ± SEM of two independent experiments with 8–10 mice/group. *, p < 0.05 vs. control mice vaccinated with calnexin and IFA alone and **, p < 0.05 vs. control mice vaccinated with soluble calnexin alone. Cytokines from lymph node cells stimulated *ex vivo* with calnexin were measured by ELISA **(D)**. The number indicates the n-fold change of mice vaccinated with calnexin+Bl-Eng2 vs. mice vaccinated with calnexin alone. *, p < vs. all other groups. Lung CFU were counted at day 18 post-infection when naïve mice were moribund, **(C+E)**. *, p < 0.05 vs. all other groups. Numbers reflect the n-fold change in lung CFU of mice vaccinated with calnexin and Bl-Eng2 vs. control mice vaccinated with calnexin or IFA alone. The survival of vaccinated mice was recorded for 30 days post-infection **(E)**. *, p < 0.05 vs. all other groups. At day 4 post-infection, the number of calnexin-specific CD4^+^ T cells were enumerated by tetramer staining **(F)**. Data represent the average ± SEM of tetramer positive cells from one of two independent experiments with 4–5 mice/group. *, p < 0.05 vs. all other groups. Cnx denotes calnexin.

To investigate whether Bl-Eng2 could be harnessed as a vaccine adjuvant, we performed preclinical studies in mice. We first tested whether Bl-Eng2 augments the development of vaccine Ag-specific T cells. To assess these T cell responses *in vivo*, we vaccinated mice with the pan-fungal Ag calnexin [21] and enumerated CD4^+^ T cell responses by TCR Tg 1807 cells, which are specific for calnexin [28]. Calnexin was suspended with incomplete freund’s adjuvant (mineral oil) and injected subcutaneously. The addition of Bl-Eng2 into the formulation sharply increased the frequency of IL-17 producing 1807 T cells (Fig 4A) and the number of activated (CD44^+^) and IL-17 and IFN-γ producing 1807 T cells, as measured by *ex vivo* stimulation with anti-CD3 and anti-CD28 mAb (Fig 4B and S4A Fig). *Ex vivo* stimulation with the vaccine Ag calnexin also yielded sharp increases in the amount of IL-17 produced by T cells from the draining lymph nodes (Fig 4D). Thus, Bl-Eng2 promoted the development of Th17 cells more so than Th1 cells.

Addition of Bl-Eng2 to the vaccine also reduced lung CFU as early as four days after mice received a lethal experimental challenge, and did so in a concentration-dependent manner (S4B Fig). In a parallel group, at the time unvaccinated control mice were moribund (day18 post-infection), the addition of Bl-Eng2 to the vaccine reduced lung CFU by more than two logs (Fig 4C). Combining the vaccine with commercial alum as an adjuvant did not increase the frequency and numbers of cytokine producing T cells or reduce the fungal burden (Fig 4A–4D). However, combining Bl-Eng-2 together with Alum increased the adjuvancy of Alum as measured by the number of activated (CD44^+^), IL-17 and IFN-γ producing 1807 T cells and the reduction in lung CFU (S5 Fig). These results suggest that Bl-Eng-2 can work in concert with other (commercially available and FDA approved) adjuvants and augment vaccine efficacy.

Bl-Eng2 failed to increase the development of Th17 and Th1 cells, the production *ex vivo* of IL-17 and IFN-γ, or reduce lung CFU in Dectin-2^-/-^ mice, verifying that the adjuvant effect is Dectin-2-dependent *in vivo* (S4C–S4E Fig). Thus, Bl-Eng2 exhibits adjuvant-like properties by increasing the development of Ag-specific (1807) Th17 and Th1 cells and protecting mice from lethal pulmonary infection with *B*. *dermatitidis*.

The studies above exploited TCR Tg T cells to sensitively report the ability of Bl-Eng2 to enhance development of calnexin Ag-specific Th17 and Th1 cells upon vaccination. However, adoptive transfer of these cells into mice artificially enhances the number of CD4^+^ T cell precursors in the animal. To investigate whether Bl-Eng2 also has the capacity to induce the development of endogenous calnexin-Ag specific CD4^+^ T cells and similarly protect animals, we vaccinated wild type mice in the absence of adoptive transfer. The formulation of Bl-Eng2 with the calnexin subunit vaccine again reduced lung CFU by over two logs vs. control mice vaccinated with calnexin in mineral oil alone (IFA), and by over 3 logs vs. mice that got IFA alone (Fig 4E). The addition of Bl-Eng2 to the calnexin vaccine formulation also increased survival significantly vs. control mice vaccinated with calnexin alone (Fig 4E). This is remarkable since the number of Ag-specific T cell precursors before vaccination was far lower in the absence than in the presence of transferred of naïve 1807 cells, indicating that Bl-Eng2 is a powerful adjuvant that drives the development of protective endogenous calnexin-specific CD4^+^ T cells (Fig 4F).

### Bl-Eng-2 augments *in vivo* killing of fungi by neutrophils (PMN) and alveolar macrophages

**Fig 5:**
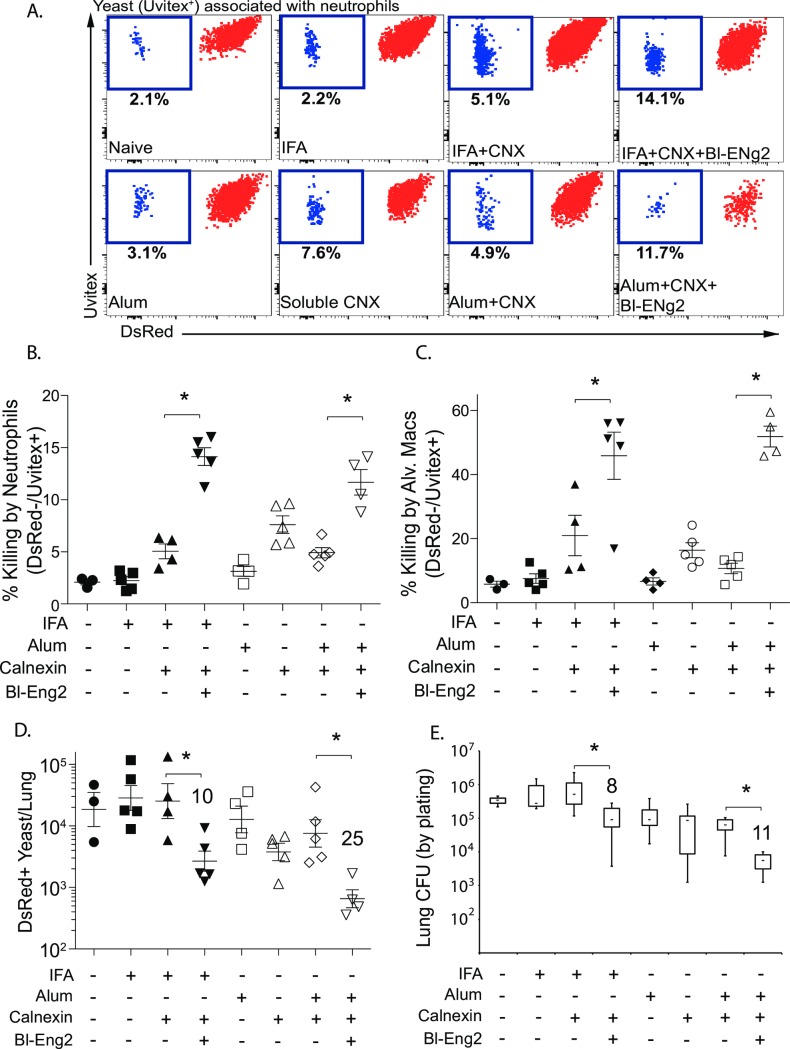
Myeloid effector mechanisms by Bl-Eng-2. Mice received 1807 cells prior to vaccination and were vaccinated and boosted with indicated adjuvants and formulated calnexin. Two weeks after the boost, mice were challenged i.t. with 10^5^ DsRed yeast and lungs were harvested 3 days later. The percentage of dead (DsRed^-^Uvitex^+^)(blue) yeast among total neutrophil-associated yeast (all Uvitex^+^ events)(blue and red together) (see gating strategy in S6A Fig) were analyzed and calculated (dot plots are concatenates from 5 mice/group) to depict the amount of killing by neutrophils **(A+B)**. The percentage of killing by alveolar macrophages is shown in (**C**). The number of live yeast was depicted by showing the total number of DsRed^+^ events **(D)** or plating lung CFU **(E)**. The numbers indicate the n-fold reduction in live yeast (DsRed^+^ or CFU) vs. the calnexin control groups. *p<0.05 control groups without Bl-Eng2. Cnx denotes calnexin.

To investigate the downstream myeloid effector mechanisms of Bl-Eng-2 adjuvancy we used red fluorescent *B*. *dermatitidis* yeast to report phagocytic uptake and fungal viability during cellular interactions with the murine leukocytes. The concept of using fluorescence to monitor microbial fate and investigate functional outcomes of individual microbial cell-host cell encounters has been introduced recently [29] and provides a powerful strategy to measure effector mechanisms *in vivo*. At day 4 post-infection, mice vaccinated with calnexin+Bl-Eng-2 and calnexin+Alum+Bl-Eng-2 showed increased activation and killing by neutrophils and alveolar macrophages vs. calnexin and calnexin+ Alum controls (Fig 5A–5C and S6 Fig). The increase in *in vivo* fungal killing by neutrophils and macrophages correlated with reduced numbers of DsRed^+^ yeast in the lung (Figs 5D and S6D) and CFU by plating (Figs 5E and S6D). Bl-Eng-2 mediated effects were observed in the presence of adoptively transferred 1807 T cells (Fig 5) and by endogenous CD4^+^ T cells without adoptive transfer (S6C and S6D Fig). Thus, the addition of Bl-Eng-2 augments the activation and killing by myeloid effector cells such as the neutrophils and alveolar macrophages in the lung.

## Discussion

We describe a novel ligand for Dectin-2: Bl-Eng2. Discovery of a potent CLR ligand may address a limitation of current vaccines: the lack of adjuvants that elicit protective cell-mediated immunity. The approach we took to identify Bl-Eng2 was based on prior work from our group and other laboratories. Dectin-2 recognizes and mediates host defense against several fungi including *C*. *albicans*, *C*. *glabrata*, *A*. *fumigatus*, *Malassezia spp*., *Coccidiodes posadasii*, *Histoplasma capsulatum* and *B*. *dermatitidis* [17,18,19,23,24,25,26,30]. Additionally, Dectin-2^-/-^ mice vaccinated with attenuated *B*. *dermatitidis* yeast fail to prime Ag-specific Th1 and Th17 cells or acquire vaccine resistance to pulmonary infection. Thus, Dectin-2 regulates innate recognition of the fungal vaccine, and the development of a protective cellular immune response [19]. Hence, we sought to identify the Dectin-2 ligand from the vaccine strain. We hypothesized that the ligand would prime APC to produce cytokines (e.g. IL-6) that are known to foster the development of Th17 cells that protect against lethal fungal challenge [6].

By using Dectin-2 reporter cells as a probe, we enriched and identified Bl-Eng2 by ConA binding, gel filtration and Mass spectrometry. The identification of Bl-Eng2 also led us to unveil the unappreciated role of Asp-Eng2 in binding Dectin-2. Both Eng2 proteins are bona fide Dectin-2 ligands since they trigger NFAT signaling in Dectin-2 reporter cells. Bl-Eng2 features a 45.2% overall and 60.1% GH16 domain sequence similarity to Eng2 from *A*. *fumigatus* (Asp-Eng2) and contains a Ser/Thr-rich C-terminus that both proteins have in common. Bl-Eng2 and Asp-Eng2 respectively harbor 68 and 74 potential *O*-linked glycosylation sites within their respective 134-aa and 234-aa long Ser/Thr-rich C-terminus, but display no consensus sites for *N*-linked glycosylation (Asn-X-Ser/Thr). In addition to the Eng2 glycoproteins, we now also establish here that MP98 from *C*. *neoformans* serves as a ligand for Dectin-2.

Dectin-2 has been reported to recognize high mannose structures of fungi [23], such as α-1,2-mannan from *C*. *albicans* [13,14] and furfurman, which is a mannoprotein from *Malassezia spp*. [25]. Man-Lam from *M*. *tuberculosis* consists of four components: a mannosyl-phophatidyl-myo-inositol (MPI) anchor, a mannose backbone, an arabinan domain, and a α1,2-mannose cap [20]. MP98 from *C*. *neoformans* is a mannoprotein with a *Mr* of 98 kDa; it contains 12 possible *N*-linked glycosylation sites, and 103 Ser/Thr residues at the C-terminus that serve as potential *O*-linked glycosylation sites [27]. The minimal unit of Bl-Eng2 that confers ligand activity is uncertain. Since both mannosidase and proteinase K digestion of CWE starting material reduced Dectin-2 ligand activity, both the protein and glycan moieties of Bl-Eng2 may contribute to its action, perhaps explaining its superior stimulation of cytokine responses compared to the other ligands.

We found that recombinant Bl-Eng2 elicits potent downstream functions. It induces the production of IL-6 by BMDC in a Dectin-2- and Card9-dependent manner. In addition, Bl-Eng2 induces the production of IL-6 and IL-1β by human PBMC, which may have strong implications for the translational aspect of our discovery. In comparison to previously described Dectin-2 ligands, Bl-Eng2 triggers superior cytokine production by murine BMDC. Ligand induced IL-6 production was >100 fold higher for Bl-Eng2 than the other Dectin-2 ligands: Furfurman from *Malassezia* spp. [25] and Mannose-capped lipoarabinomannan (Man-Lam) from *M*. *tuberculosis* [20] and MP98 from *C*. *neoformans* [27].

Bl-Eng2 induction of T cell priming cytokines by APCs efficiently promoted the development of calnexin Ag-specific Th17 cells (more so than Th1 cells), and recall of these cells to the lung upon fungal challenge of vaccinated mice. The large numbers of pro-inflammatory T cells sharply reduced lung CFU and increased survival after infection of Bl-Eng2 vaccinated vs. control mice. In comparison, combining commercial Alum with the calnexin subunit vaccine did not show an adjuvant effect. However, Bl-Eng-2 combined with Alum augmented its adjuvancy indicating that Bl-Eng-2 has the potential to improve T cell priming by the commercially available and FDA approved Alum. Thus, in our subunit vaccine model, Bl-Eng2-induced Dectin-2 signaling was associated with cellular immune responses that protected mice against lethal pulmonary fungal infection. Although not experimentally addressed in this manuscript, it is conceivable that Bl-Eng-2 can also augment the induction of CD4^+^ T cell-dependent antibody responses that promote host protection against fungi, especially when combined with Alum since the latter is known to stimulate both T and B cell immune responses [31]. It remains to be investigated whether antibody will be protective in our vaccine setting [32].

We previously reported that mice vaccinated with calnexin and other adjuvants (glucan particles engaging Dectin-1, Adjuplex, or the combination) were optimally protected when we adoptive transferred naïve 1807 cells to increase the pool of Ag-experienced CD4^+^ T cells [21]. Here, the addition of Bl-Eng2 to the same calnexin vaccine reduced lung CFU by more than two to three logs vs. control mice even without adoptive transfer of large numbers of naïve 1807 T cell precursors. These results imply that engagement of Dectin-2 by Bl-Eng2 may be better than engagement of Dectin-1 by glucan particles and other previously used adjuvants at expanding the pool of endogenous calnexin-specific CD4^+^ T cell precursors or that Bl-Eng2 induced individual Ag-experienced cells to produce larger amounts of effector cytokines. Thus, Bl-Eng2 may be a powerful vaccine adjuvant in situations where T cell precursors are low in number and adoptive transfer of naïve T cell precursors is either not feasible or too costly.

In contrast to the protective effects of Bl-Eng2 vaccination, Man-Lam induced Dectin-2 responses that caused Th17 cell-mediated autoimmune disease pathology and EAE [20]. Man-Lam stimulation of Dectin-2 led to the development of MOG_35-55_ peptide-specific T cells that produced IL-17, IFN-γ and GM-CSF upon *ex vivo* stimulation. This could simply relate to model selection rather than adjuvant efficiency. Thus, it is unclear whether Man-Lam is capable of inducing protective T cell immunity in an infectious disease setting. Although *C*. *neoformans* MP98 and its glycan modifications also promoted T cell activation, the T-helper phenotype and functional role in resistance by primed T cells were not investigated [33,34].

In conclusion, among the few Dectin-2 ligands reported to date, or newly discovered here, Bl-Eng2 is the most potent at stimulating murine and human cells to produce cytokines known to foster the development of protective Th17 and Th1 cells e.g. IL-6 and IL-1β. The production of IL-17 and IFN-γ by Th17 and Th1 cells then promotes the activation and killing of fungi by myeloid effector cells such as neutrophils and alveolar macrophages [6]. Since Bl-Eng2 also greatly augments protective immunity mediated by a subunit vaccine, Bl-Eng2 could potentially be harnessed as an adjuvant for vaccination against infectious disease that requires cellular immunity for host defense. The structural basis underpinning Bl-Eng2 potency as an adjuvant will be important to investigate and understand so that those features can be harnessed for vaccine development in the fight against infectious disease due to intracellular pathogens.

## Material and methods

### Fungi

Strains used were wild-type, virulent *B*. *dermatitidis* ATCC strain 26199, DsRed26199 [35] and strain #55, the isogenic, attenuated mutant lacking BAD1 [36]. *B*. *dermatitidis* was grown as yeast on Middlebrook 7H10 agar with oleic acid-albumin complex (Sigma) at 39°C.

### Mouse strains

Inbred wild type C57BL/6 and congenic B6. PL-Thy1^a^/Cy (stock #00406) mice carrying the Thy 1.1 allele were obtained from Jackson Laboratories, Bar Harbor, ME. *Blastomyces*-specific TCR Tg 1807 mice were generated in our lab and were backcrossed to congenic Thy1.1^+^ mice as described elsewhere [28]. Dectin-2^-/-^ [14] mice were bred at our facility. Mice were 7–8 weeks old at the time of these experiments. Mice were housed and cared for as per guidelines of the University of Wisconsin Animal Care Committee who approved all aspects of this work.

### Preparation of CWE

*Blastomyces dermatitidis* yeast were harvested from 7H10 agar, washed with H_2_O, and sonicated for 3 min on ice. After centrifuging, the soluble extract was collected, passed through a 0.45-μm pore-size filter and used as CWE. The protein level was measured with the Pierce BCA assay (Thermo Fisher Scientific).

### Enrichment of mannosylated proteins and mass spectrometry analysis

To enrich the mannosylated proteins, CWE was incubated with Concanavalin A (ConA) Sepharose resin (Sigma-Aldrich), and the bound fraction was eluted with methyl-α-D-mannopyranoside as described previously [21]. The ConA-enriched proteins were then applied to a size exclusion column of Ultragel AcA 44 resin (Pall) and eluted with PBS. The ConA enrichment and size exclusion fractions were assessed using SDS-PAGE and silver staining. Size exclusion fractions that contained Dectin-2 ligand activity were analyzed by mass spectrometry as previously described [21] at the Mass Spectrometry Facility, University of Wisconsin-Madison. Briefly, peptides were analyzed by nano-LC-MS/MS using the Agilent 1100 nanoflow system (Agilent Technologies) connected to a hybrid linear ion trap-orbitrap mass spectrometer (LTQ-Orbitrap XL, Thermo Fisher Scientific) equipped with a nanoelectrospray ion source.

### Generation and purification of r-Bl-Eng2

Bl-Eng2 was cloned and expressed in *P*. *pastoris* using standard recombinant techniques. Total RNA was extracted from *B*. *dermatitidis* yeast and transcribed to cDNA as previously described [37]. Using the cDNA as a template, the *Bl-ENG2* coding sequence was amplified using KOD Hot Start DNA Polymerase (Toyobo) with primers 5′-GGCTCGAGAAAAGAGAGGCTGAAGCTAGGGCTACCAAGCTCGCGTT and 5′-GTTTCTAGACCGTACTTGTCATTTGTGGGGTATCCCG, and inserted in-frame into the *Xho*I/*Xba*I sites of the pPICZαA vector (Invitrogen). The resulting expression vector was then linearized with *Pme*I and transformed into *Pichia pastoris* strain X-33 (Invitrogen) by electroporation. Yeast colonies were screened for Bl-Eng2 protein expression by Western blot analysis using an anti-His antibody (Cell Signaling Technology). Bl-Eng2 protein secreted from methanol-induced yeast cells was purified using Ni-NTA agarose (Qiagen) according to the manufacturer's protocol, and dialyzed against PBS. Purity of recombinant Bl-Eng2 was assessed by SDS-PAGE and silver staining.

### Carbohydrate analysis

Bl-Eng2 protein glycosylation was assessed using the Pierce Glycoprotein Staining Kit (Thermo Fisher Scientific). Monosaccharide composition was determined by gas chromatography as described elsewhere [38].

### CLR reporter assay

B3Z/BWZ reporter cells expressing Dectin-2, Mincle, MCL and Dectin-1 have been described previously [19,39]. For B3Z/BWZ cell stimulation, 10^5^ B3Z/BWZ cells per well in a 96-well plate were incubated for 18 h with heat-killed fungal cells or plate-coated ligands. β-galactosidase (lacZ) activity was measured in total cell lysates using CPRG (Roche) as a substrate. OD 560 was measured using OD 620 as a reference.

### Stimulation of mouse BMDCs or human PBMCs and cytokine detection

Generation of bone marrow–derived dendritic cells (BMDCs) has been described previously [19]. Peripheral blood mononuclear cells (PBMCs) were isolated from heparinized whole blood collected over Ficoll-Paque Plus (GE). 1–2 × 10^5^ BMDCs or 5 × 10^5^ PBMCs per well in a 96-well plate were incubated with plate-bound Bl-Eng2. After 24 h, supernatants were collected and cytokine levels were measured by ELISA (R&D Systems or Biolegend) according to the manufacturer’s specifications.

### Vaccination with Calnexin and Bl-Eng2 and enumeration of rare epitope-specific T cells

Prior to vaccination, mice received adoptively transferred naïve 1807 T cells [28] or not. Mice were vaccinated twice subcutaneously with 10μg recombinant calnexin and 10μg Bl-Eng2 formulated in incomplete Freund’s adjuvant (IFA), two weeks apart. Two weeks after the boost, mice were challenged with 2x10^4^ 26199 yeast and analyzed for lung T cell responses (at day 4 post-infection) and lung CFU (at day 4 or two weeks post-infection). 1807 T cell responses were detected with the congenic Thy1.1 marker and endogenous, calnexin-specific T cells by tetramer [28]. T cells were detected using the following antibodies: tetramer-PE, CD4-BUV395, CD8-PeCy7, CD3-BV421, CD90.2-BV785, CD44-BV650, Live-dead Near IR, IFN-γ-A488 and IL-17-A647.

### Intracellular cytokine stain

Lung cells were harvested at day 4 post-infection. Cells (0.5 × 10^6^ cells/ml) were stimulated for 5 hours with anti-CD3 (clone 145-2C11; 0.1μg/ml) and anti-CD28 (clone 37.51; 1μg/ml) in the presence of Golgi-Stop (BD Biosciences). Stimulation with fungal ligands yielded comparable cytokine production by transgenic T-cells compared to CD3/CD28 stimulation. After cells were washed and stained for surface CD4 and CD8 using anti-CD4 BV395, anti-CD8 PeCy7, and anti-CD44-FITC mAbs (Pharmingen), they were fixed and permeabilized in Cytofix/Cytoperm at 4°C overnight. Permeabilized cells were stained with anti-IL-17A PE and anti-IFN-γ Alexa 700 (clone XMG1.2) conjugated mAbs (Pharmingen) in FACS buffer for 30 min at 4°C, washed, and analyzed by FACS. Cells were gated on CD4 and cytokine expression in each gate analyzed. The number of cytokine positive CD4^+^ T cells per lung was calculated by multiplying the percent of cytokine- producing cells by the number of CD4^+^ T cells in the lung.

### The generation of bone marrow dendritic cells

Bone marrow-derived dendritic cells (BMDCs) were obtained from the femurs and tibias of individual mice. Each bone was flushed with 10 ml of 1% FBS in RPMI through a 22G needle. Red blood cells were lysed followed by wash and re-suspension of cells in 10% FBS in RPMI medium. In a petri dish, 2 × 10^6^ bone marrow cells were plated in 10 ml of RPMI containing 10% FBS plus penicillin-streptomycin (P/S) (HyClone), 2-mercaptoethanol and 20 ng/ml of rGM-CSF. The culture media were refreshed every three days and BMDCs were harvested after 10 days for *in vitro* co-culture assays.

### *Ex vivo* stimulation of primed T cells for cytokine protein measurement

*Ex vivo* cell culture supernatants were generated using the brachial and inguinal draining lymph nodes harvested from mice 28 days post-vaccination and at day 4 post-infection, washed and resuspended in complete RPMI containing 10 μg/ml recombinant calnexin [21,40], and plated in 96-well plates at a concentration of 5 × 10^5^ cells/well. Supernatants were collected from *ex vivo* co-cultures after three days of incubation at 37°C and 5% CO_2_ [6]. IFN-γ and IL-17 (R&D System) were measured by ELISA according to manufacturer specifications (detection limits, 0.05 ng/ml and 0.02 ng/ml, respectively).

### Tracking association of yeast with neutrophils and alveolar macrophages *in vivo*

Mice were euthanized three days after challenge i.t. with 10^5^ DsRed yeast and hearts were perfused with PBS to remove blood from the lungs to improve staining. Lungs were dissociated, digested and stained as described previously [35]. In summary, lungs were dissociated and digested in buffer containing collagenase D and DNase I. After erythrocyte lysis, cells were stained for myeloid cell markers and then fixed in Cytofix/Cytoperm (BD Biosciences, San Jose, CA). Cells were stained for 30 minutes at room temperature with 1 μg/ml Uvitex-2B (Polysciences, Warrington, PA) diluted in BD perm/wash buffer and then subsequently washed with BD perm/wash buffer and fixed with 2% paraformaldehyde.

### Statistics

Differences in the number of cells and lung CFU were analyzed using Wilcoxon rank and Mann Whitney test for non-parametric data or a T-test if data were normally distributed. A Bonferroni adjustment was used to correct for multiple tests. A value of *P* < 0.05 is considered significant.

### Ethics statement

Studies with human peripheral blood mononuclear cells were approved by University of Wisconsin-Madison IRB (protocol 2014–1167 CR002) and patients provided informed written consent.

The animal studies performed were governed by protocols M00969 as approved by the IACUC committees of the University of Wisconsin-Madison Medical School. Animal studies were compliant with all applicable provisions established by the Animal Welfare Act and the Public Health Services (PHS) Policy on the Humane Care and Use of Laboratory Animals.

## Supporting information

S1 Fig Separation, characterization, and enrichment of Dectin-2 ligand activity. **(A)** 100 μg CWE was fractionated by a GELFREE (GF) 8100 system. The fractions were separated by SDS-PAGE and silver stained. **(B)** Acetone-precipitated fractions were assayed for ligand activity. **(C)** Fractions 4–6 from the 1^st^ gel filtration contained most of the ligand activity (see Fig 1F); they were separated by a second run over the size exclusion column (blue line represents the trace line of A_280_ absorption). Fractions were tested by Dectin-2 reporter cells for ligand activity. Fractions 9–13 contained most of the ligand activity and were determined the positive pool; fractions 1–7 were the negative pool for the subsequent mass spec analysis.
(TIFF)

S2 Fig Mass spec analysis identifies Bl-Eng2 as a candidate ligand for Dectin-2. **(A)** Complete list of Mass spec candidates for Dectin-2 ligands. **(B)** Amino acid sequence of recombinant Bl-Eng2 contains 637 amino acids. Colored aa match the protein domains illustrated in Fig 2B.
(TIFF)

S3 Fig *Aspergillus* Eng2 is a Dectin-2 ligand. **(A)** 0.6 ug *Pichia*-expressed *Aspergillus* Eng-2 was plate-coated and tested for ligand activity using CLR expressing B3Z and BWZ reporter cells. **(B)** 30 ng plate-coated *Pichia*-expressed *Blastomyces* Eng2 and *Aspergillus* Eng2 was tested for ligand activity with Dectin-2 expressing B3Z reporter cells. **(C)** 30 ng plate-coated *Pichia-*expressed *Cryptococcus* Eng2 was tested for ligand activity with Dectin-2 expressing B3Z reporter cells. **(D)** Supernatants from BMDCs (2 × 10^5^ per well) co-cultured with plate-coated MP98 were analyzed for IL-6 by ELISA.
(TIFF)

S4 Fig Bl-Eng2 induces the development of Th17 and Th1 cells in a Dectin-2 dependent manner and reduces lung CFU concentration dependently. **(A+C)** Mice were subcutaneously vaccinated twice with calnexin and Bl-Eng2, two weeks apart and challenged intratracheally with *B*. *dermatitidis* 26199 yeast two weeks post-vaccination. At day 4 post-infection, the numbers of activated (CD44^+^) and cytokine producing 1807 T cells in wild type **(A)** and Dectin-2^-/-^ mice **(C)** were enumerated by FACS. Data represent the average ± SEM of 5 mice/group. *, p < 0.05 vs calnexin-vaccinated control mice. Lymph node (LN) cells from the draining brachial LN were stimulated *ex vivo* with calnexin and cytokines in the cell culture supernatants were measured by ELISA **(D)**. **(B+E)** At day 4 post-infection, lung CFU of **(B)** wild type mice and **(E)** Dectin-2^-/-^ mice were determined by plating lung homogenates. *, p < 0.05 vs calnexin-vaccinated control mice. **(A-E)** Numbers reflect the n-fold change of mice vaccinated with calnexin and Bl-Eng2 vs. control mice vaccinated with calnexin. NS; not statistically significant.
(TIFF)

S5 Fig Bl-Eng2 augments adjuvancy of Alum. **(A-C)** Mice were subcutaneously vaccinated with 5μg calnexin and 10μg Bl-Eng2 or/and alum twice, two weeks apart, and then challenged intratracheally with *B*. *dermatitidis* 26199 yeast two weeks post-vaccination. At day 4 post-infection, the numbers of activated (CD44^+^) and cytokine-producing 1807 cells in the lung were enumerated by FACS **(A+B)**. Data represent the average ± SEM of 5 mice/group. *, p < 0.05 vs. control mice vaccinated with calnexin and Alum. The numbers indicate the n-fold change of mice vaccinated with Alum+calnexin+Bl-Eng2 vs. mice vaccinated with Alum+calnexin. *, p < vs. all other groups. Lung CFU were counted at day 4 post-infection **(C)**. The numbers indicate the n-fold change in lung CFU of mice vaccinated with Alum+calnexin+Bl-Eng2 vs. mice vaccinated with Alum+calnexin. *, p < 0.05 vs. all other groups. Cnx denotes calnexin.
(TIFF)

S6 Fig Gating strategy for tracking neutrophil- and alveolar macrophage-associated with yeast, activation of PMN and myeloid effector killing in the absence of 1807 T cell transfer. Viable cells (negative for fixable live/dead dye) that were Siglec F^-^, CD11b^+^, Ly6G^+^ and Ly6C^int^ gated as neutrophils (PMNs) and SiglecF^+^, CD11c^+^ gated as alveolar macrophages **(A)**. *Blastomyces* yeast have higher side scatter than most leukocytes, so Uvitex^+^, SSC^hi^ neutrophils are associated with yeast. Phagocytes in the lungs that have phagocytosed inhaled chitin (from bedding/food) stain with Uvitex when cells are permeabilized. The cells that have phagocytosed chitin/cellulose have decreased Uvitex fluorescence but tend to be autofluorescent in many channels including DsRed; an additional gate was placed on Uvitex^+^ events to remove any false positives in the neutrophil gate. Activated (CD11b^hi^) neutrophils from the neutrophil gate were calculated and shown in panel **(B)**. Myeloid effector killing in the absence of 1807 T cells **(C+D)**. Mice did not receive adoptive transfer of 1807 cells prior to vaccination and were vaccinated twice with calnexin +/- Bl-Eng-2 emulsified in IFA. Two weeks after the boost, mice were challenged i.t. with 10^5^ DsRed yeast and lungs were harvested 3 days later. The percentage of dead (DsRed^-^Uvitex^+^)(blue) among total neutrophil- or macrophage-associated yeast (all Uvitex^+^ events)(blue and red together) (see gating strategy in S6A Fig) were analyzed and calculated (dot plots are concatenates from 5 mice/group) to depict the amount of killing by PMN and macrophages **(C)**. The number of live yeast was depicted by showing the total number of DsRed^+^ events or plating lung CFU **(D)**. The number indicates the n-fold reduction in lung CFU vs. the calnexin control group. *p<0.05 control groups without Bl-Eng-2.
(TIFF)
