# Supplementary figures and images for: Ligation of Dectin-2 with a novel microbial ligand promotes adjuvant activity for vaccination
Source: PLoS Pathog. 2017 Aug 9;13(8):e1006568. doi: 10.1371/journal.ppat.1006568 (PMC5565193; doi:10.1371/journal.ppat.1006568)

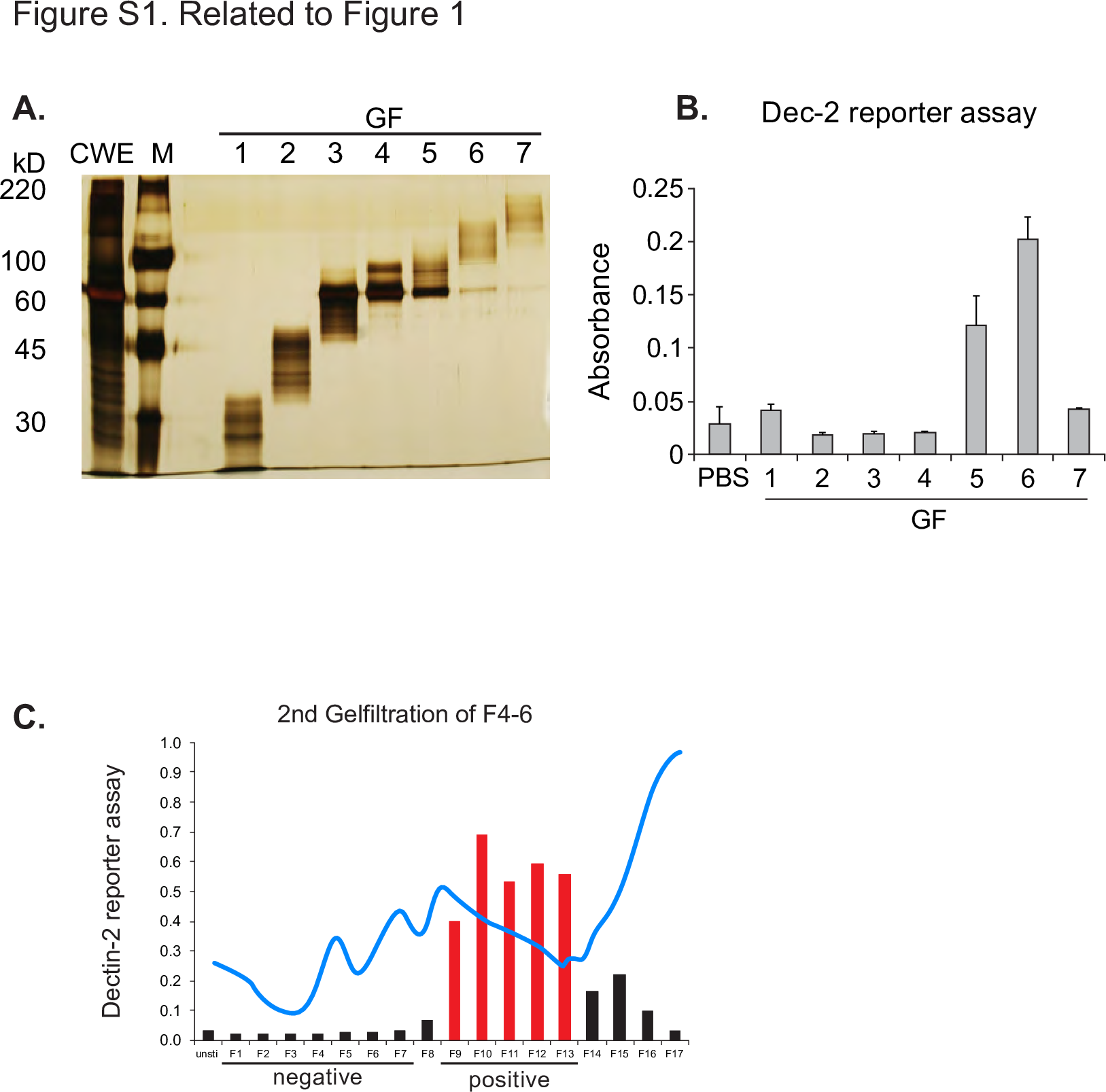

Supplement: S1 Fig — (A) 100 μg CWE was fractionated by a GELFREE (GF) 8100 system. The fractions were separated by SDS-PAGE and silver stained. (B) Acetone-precipitated fractions were assayed for ligand activity. (C) Fractions 4–6 from the 1st gel filtration contained most of the ligand activity (see Fig 1F); they were separated by a second run over the size exclusion column (blue line represents the trace line of A280 absorption). Fractions were tested by Dectin-2 reporter cells for ligand activity. Fractions 9–13 contained most of the ligand activity and were determined the positive pool; fractions 1–7 were the negative pool for the subsequent mass spec analysis. (TIFF) [file ppat.1006568.s001.tiff]

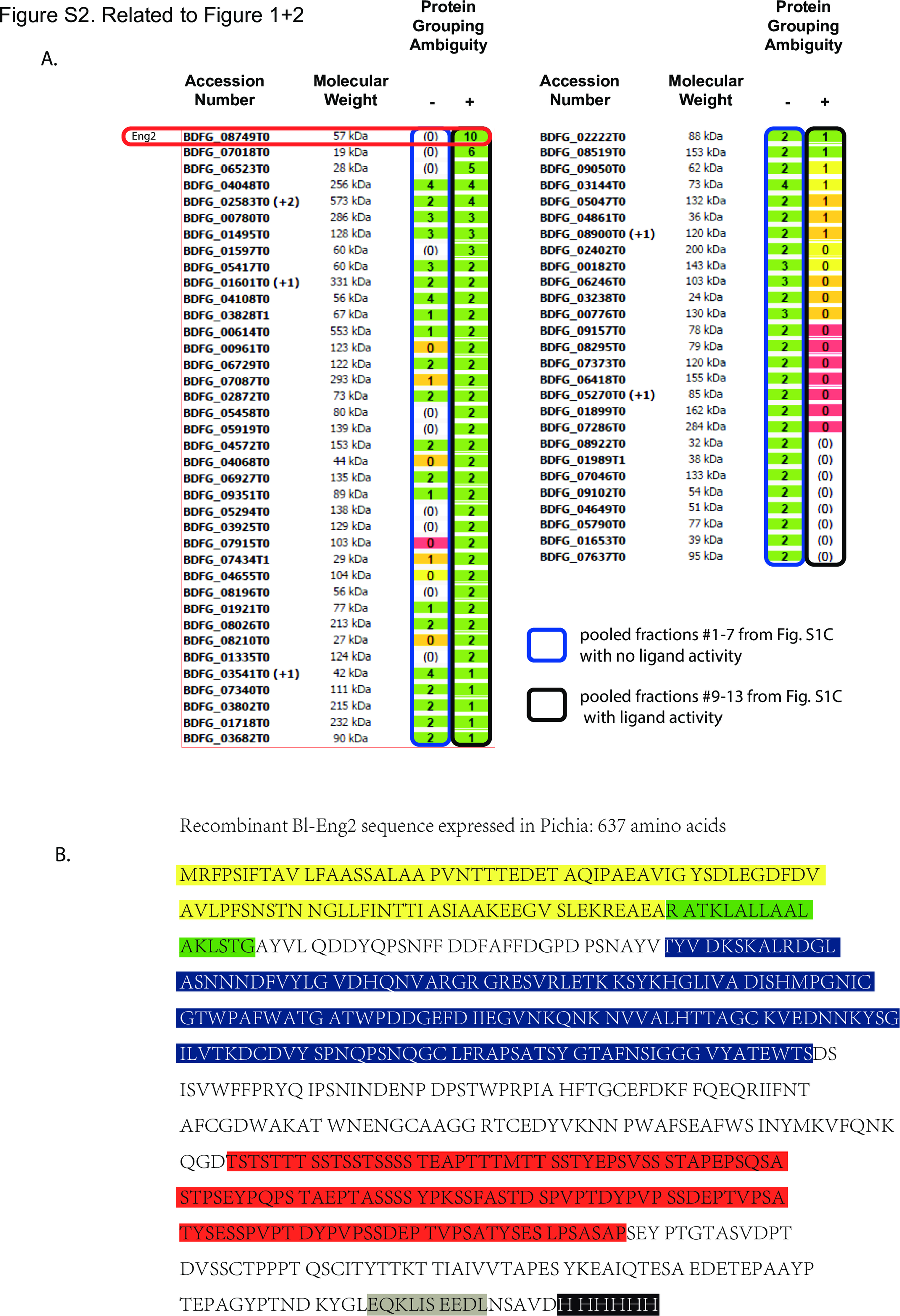

Supplement: S2 Fig — (A) Complete list of Mass spec candidates for Dectin-2 ligands. (B) Amino acid sequence of recombinant Bl-Eng2 contains 637 amino acids. Colored aa match the protein domains illustrated in Fig 2B. (TIFF) [file ppat.1006568.s002.tiff]

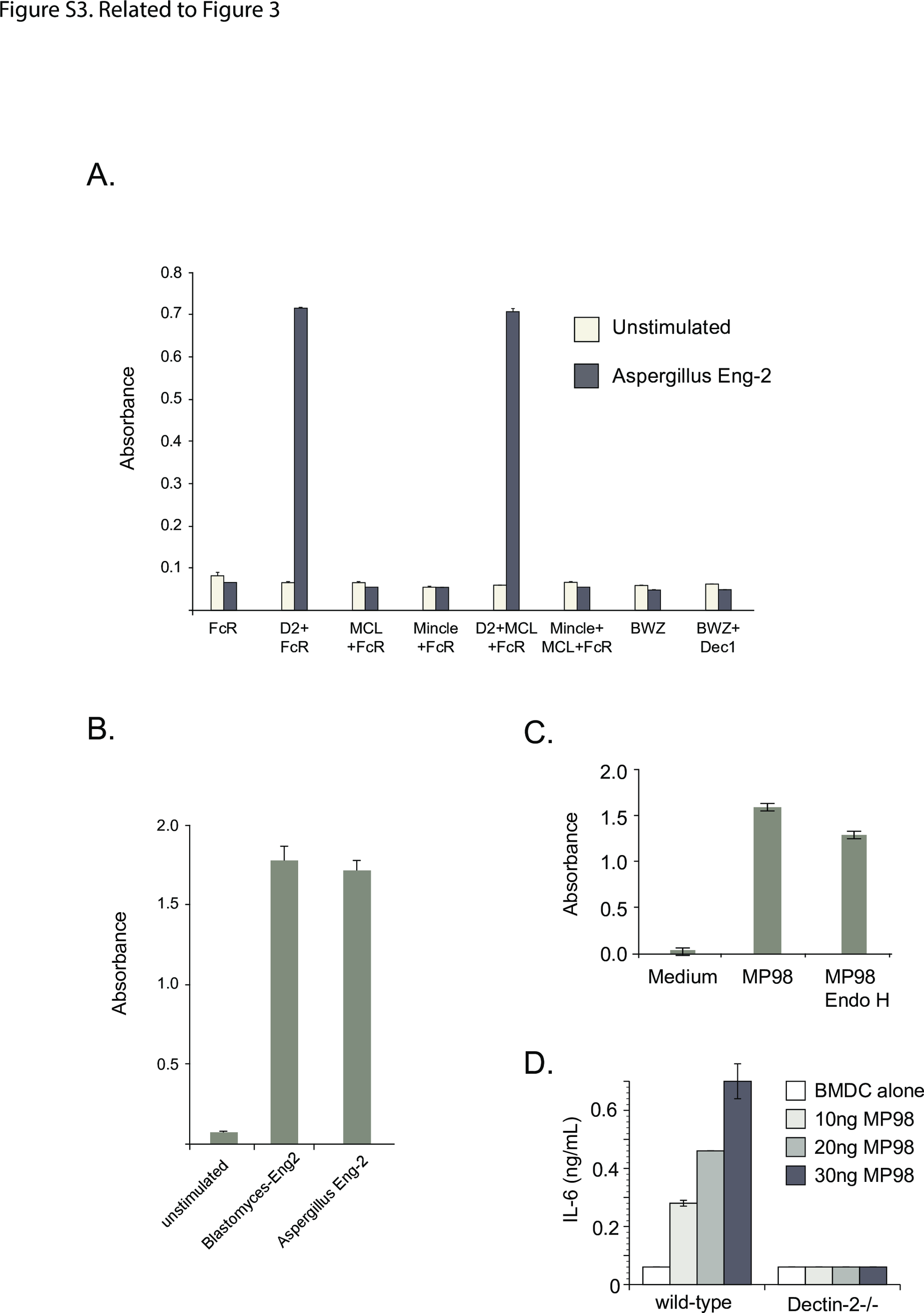

Supplement: S3 Fig — (A) 0.6 ug Pichia-expressed Aspergillus Eng-2 was plate-coated and tested for ligand activity using CLR expressing B3Z and BWZ reporter cells. (B) 30 ng plate-coated Pichia-expressed Blastomyces Eng2 and Aspergillus Eng2 was tested for ligand activity with Dectin-2 expressing B3Z reporter cells. (C) 30 ng plate-coated Pichia-expressed Cryptococcus Eng2 was tested for ligand activity with Dectin-2 expressing B3Z reporter cells. (D) Supernatants from BMDCs (2 × 10^5^ per well) co-cultured with plate-coated MP98 were analyzed for IL-6 by ELISA. (TIFF) [file ppat.1006568.s003.tiff]

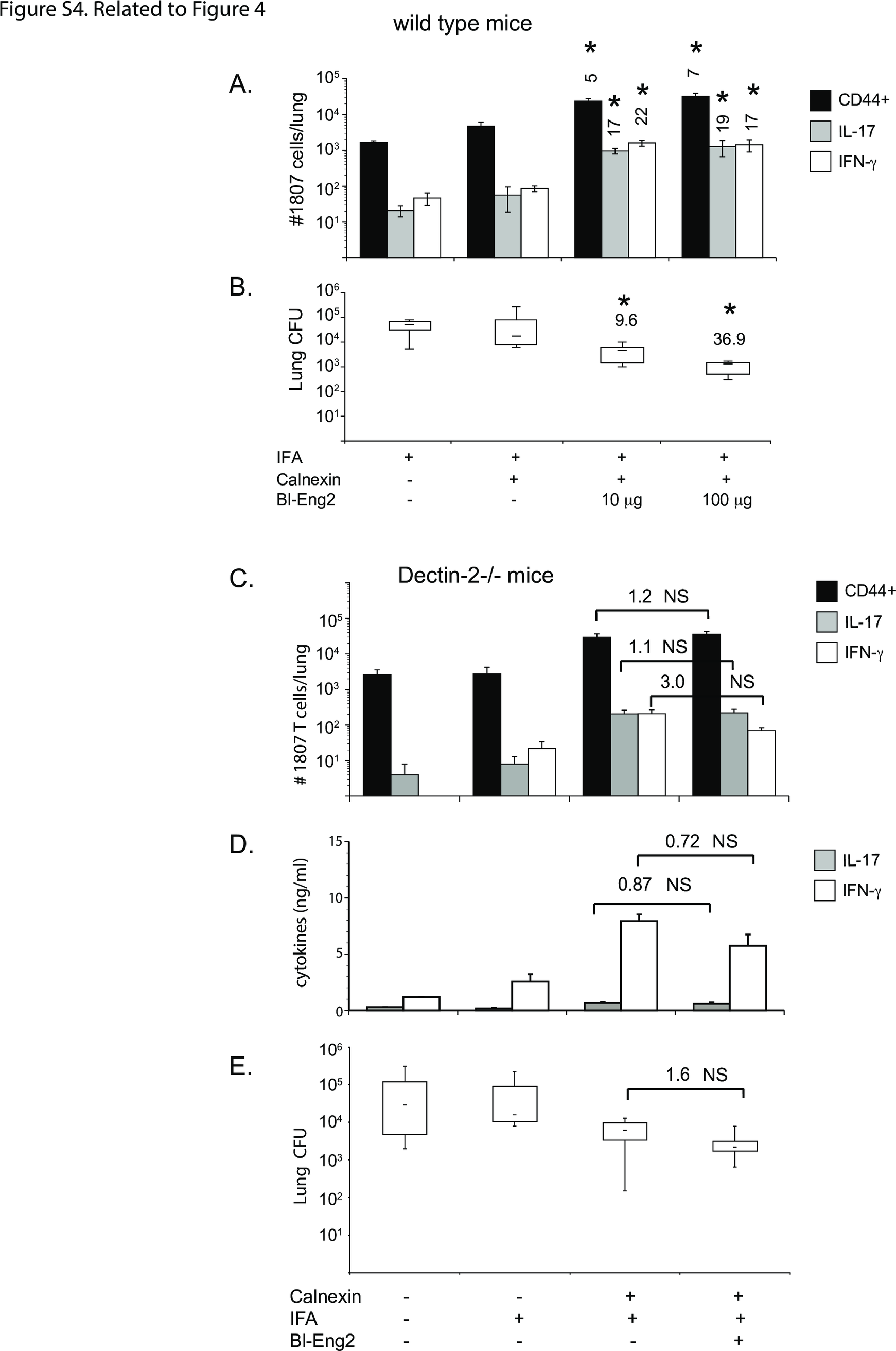

Supplement: S4 Fig — (A+C) Mice were subcutaneously vaccinated twice with calnexin and Bl-Eng2, two weeks apart and challenged intratracheally with B. dermatitidis 26199 yeast two weeks post-vaccination. At day 4 post-infection, the numbers of activated (CD44+) and cytokine producing 1807 T cells in wild type (A) and Dectin-2-/- mice (C) were enumerated by FACS. Data represent the average ± SEM of 5 mice/group. *, p < 0.05 vs calnexin-vaccinated control mice. Lymph node (LN) cells from the draining brachial LN were stimulated ex vivo with calnexin and cytokines in the cell culture supernatants were measured by ELISA (D). (B+E) At day 4 post-infection, lung CFU of (B) wild type mice and (E) Dectin-2-/- mice were determined by plating lung homogenates. *, p < 0.05 vs calnexin-vaccinated control mice. (A-E) Numbers reflect the n-fold change of mice vaccinated with calnexin and Bl-Eng2 vs. control mice vaccinated with calnexin. NS; not statistically significant. (TIFF) [file ppat.1006568.s004.tiff]

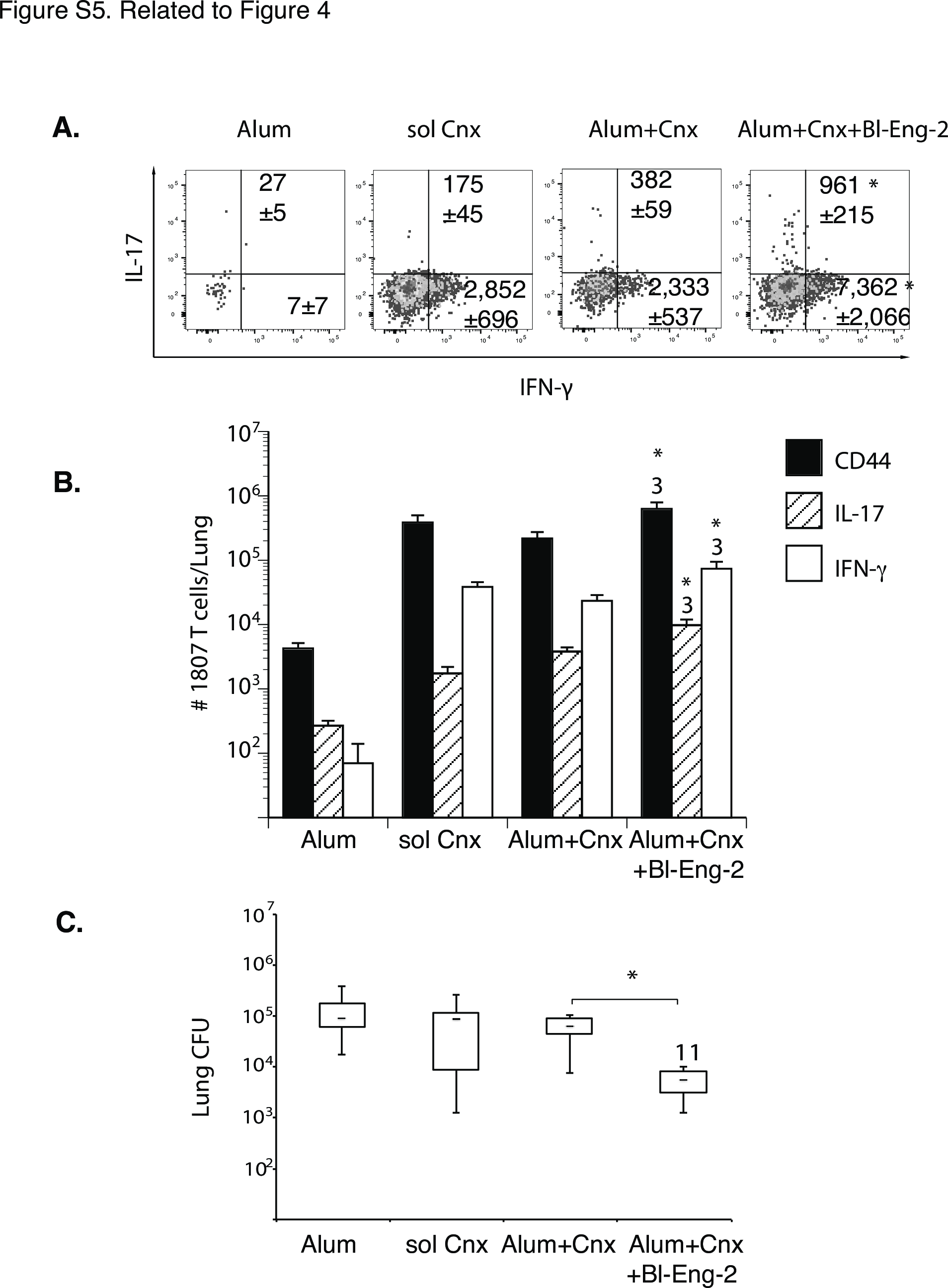

Supplement: S5 Fig — (A-C) Mice were subcutaneously vaccinated with 5μg calnexin and 10μg Bl-Eng2 or/and alum twice, two weeks apart, and then challenged intratracheally with B. dermatitidis 26199 yeast two weeks post-vaccination. At day 4 post-infection, the numbers of activated (CD44+) and cytokine-producing 1807 cells in the lung were enumerated by FACS (A+B). Data represent the average ± SEM of 5 mice/group. *, p < 0.05 vs. control mice vaccinated with calnexin and Alum. The numbers indicate the n-fold change of mice vaccinated with Alum+calnexin+Bl-Eng2 vs. mice vaccinated with Alum+calnexin. *, p < vs. all other groups. Lung CFU were counted at day 4 post-infection (C). The numbers indicate the n-fold change in lung CFU of mice vaccinated with Alum+calnexin+Bl-Eng2 vs. mice vaccinated with Alum+calnexin. *, p < 0.05 vs. all other groups. Cnx denotes calnexin. (TIFF) [file ppat.1006568.s005.tiff]

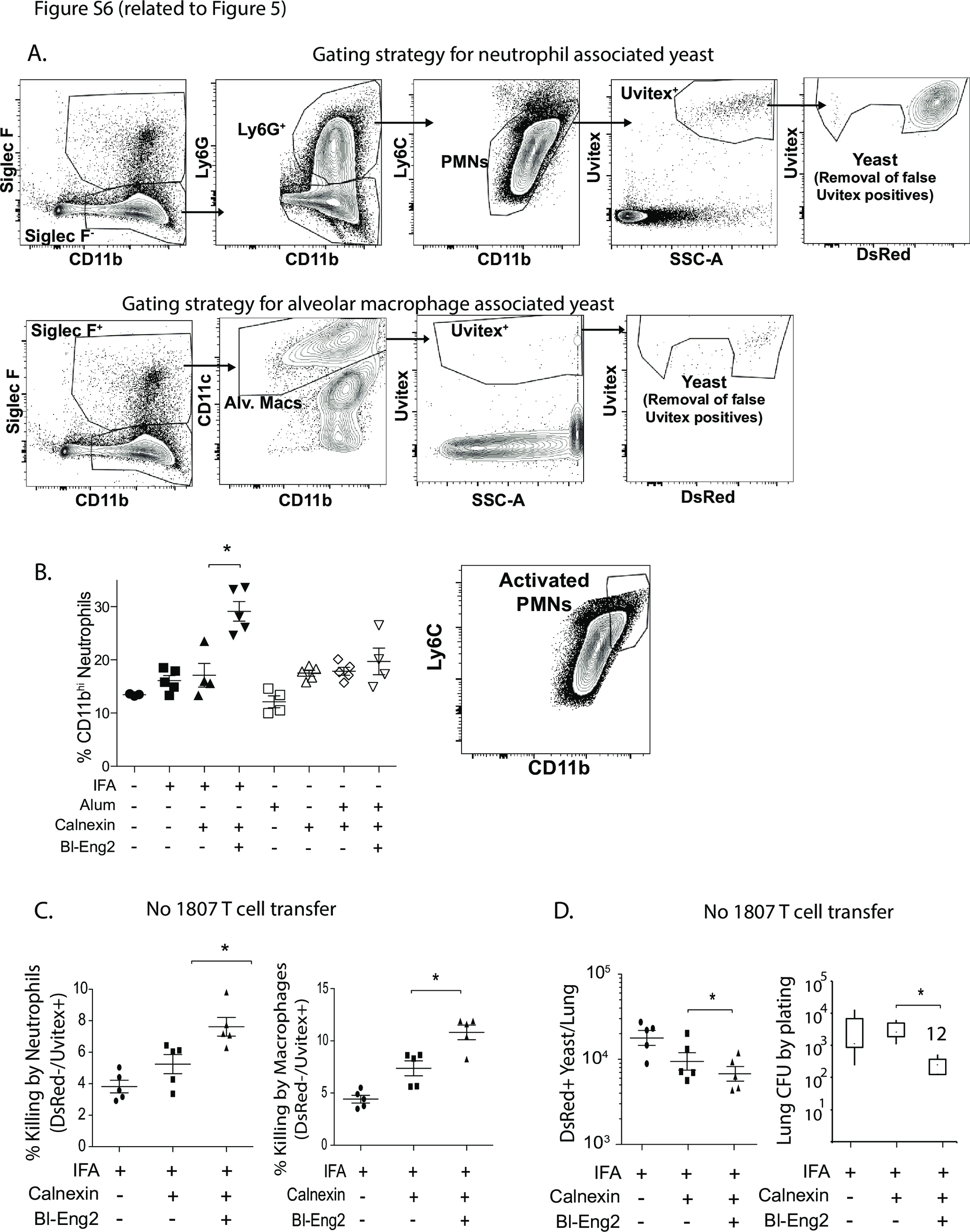

Supplement: S6 Fig — Viable cells (negative for fixable live/dead dye) that were Siglec F-, CD11b+, Ly6G+ and Ly6Cint gated as neutrophils (PMNs) and SiglecF+, CD11c+ gated as alveolar macrophages (A). Blastomyces yeast have higher side scatter than most leukocytes, so Uvitex+, SSChi neutrophils are associated with yeast. Phagocytes in the lungs that have phagocytosed inhaled chitin (from bedding/food) stain with Uvitex when cells are permeabilized. The cells that have phagocytosed chitin/cellulose have decreased Uvitex fluorescence but tend to be autofluorescent in many channels including DsRed; an additional gate was placed on Uvitex+ events to remove any false positives in the neutrophil gate. Activated (CD11bhi) neutrophils from the neutrophil gate were calculated and shown in panel (B). Myeloid effector killing in the absence of 1807 T cells (C+D). Mice did not receive adoptive transfer of 1807 cells prior to vaccination and were vaccinated twice with calnexin +/- Bl-Eng-2 emulsified in IFA. Two weeks after the boost, mice were challenged i.t. with 105 DsRed yeast and lungs were harvested 3 days later. The percentage of dead (DsRed-Uvitex+)(blue) among total neutrophil- or macrophage-associated yeast (all Uvitex+ events)(blue and red together) (see gating strategy in S6A Fig) were analyzed and calculated (dot plots are concatenates from 5 mice/group) to depict the amount of killing by PMN and macrophages (C). The number of live yeast was depicted by showing the total number of DsRed+ events or plating lung CFU (D). The number indicates the n-fold reduction in lung CFU vs. the calnexin control group. *p<0.05 control groups without Bl-Eng-2. (TIFF) [file ppat.1006568.s006.tiff]
